# Nonlinear Non-Equilibrium Thermodynamics Based on the Ehrenfest–Klein Model

**DOI:** 10.3390/e22030293

**Published:** 2020-03-02

**Authors:** Gleb A. Zhernokleev, Leonid M. Martyushev

**Affiliations:** 1 Technical Physics Department, Ural Federal University, 19 Mira St., 620002 Ekaterinburg, Russia; glebster47@mail.ru; 2 Institute of Industrial Ecology, Russian Academy of Sciences, 20 S. Kovalevskaya St., 620219 Ekaterinburg, Russia

**Keywords:** entropy, urn model, fluctuation theorem

## Abstract

Nonlinear non-equilibrium thermodynamic relations have been constructed based on the generalized Ehrenfest–Klein model. Using these relations, the behavior of the entropy and its production in time at arbitrary deviations from equilibrium has been studied. It has been shown that the transient fluctuation theorem is valid for this model if a dissipation functional is treated as the thermodynamic entropy production.

## 1. Introduction

The behavior of the entropy in nonequilibrium processes has become of interest immediately after the introduction of this function of state. The greatest scientists of the past: L. Boltzmann, A. Poincaré, J. Gibbs, L. Onsager, I. Prigogine, etc. actively studied this issue. Currently, interest in entropy and its change has been refreshed after the proof of the so-called fluctuation theorems for a number of nonequilibrium systems [1,2,3,4].

The behavior of the entropy near equilibrium is well studied within linear nonequilibrium thermodynamics. This theory, which arose initially as a generalization of experimental facts, is now firmly included in the foundation of modern science [5,6,7,8,9,10]. This is largely facilitated by its consistency not only with experiment, but also with a number of classical models (a weakly non-equilibrium rarefied gas satisfying the Boltzmann equation, etc.). However, classical linear nonequilibrium thermodynamics is inapplicable if deviations from equilibrium are arbitrary in magnitude. For this case, development of so-called nonlinear nonequilibrium thermodynamics is currently of interest. Being extremely demanding in theory and practice, nonlinear nonequilibrium thermodynamics has not yet been completed and is under active development [7,8,9,10,11,12,13]. For this reason, it is extremely interesting and important to test and match the foundations of nonlinear nonequilibrium thermodynamics with classical nonlinear statistical models. One such model was proposed at the beginning of the 20th century by Paul and Tatiana Ehrenfest [14]. This model was introduced in order to solve the irreversibility paradoxes that arose during the scientific discussion between Boltzmann on the one hand and Poincaré with like-minded scientists on the other hand. Advantages of the model is its relative simplicity, the possibility of an exact analytical solution, and its applicability to describe the relaxation of the system with an arbitrary initial deviation from equilibrium. Subsequently, this model was used as a foundation for a whole class of precisely solvable models known as urn models (or dog–flea models). These models are different in complexity and purposes. In particular, in recent works [15,16,17], diverse variations of Ehrenfest urn models are presented as a tool for studying various theories of equilibrium and nonequilibrium statistical physics. In this work, the historically first modification of the Ehrenfest model proposed by Klein [18] is considered.

The aim of this work is to develop nonlinear nonequilibrium thermodynamics based on the generalized Ehrenfest–Klein model and to apply it to study the time evolution of the entropy at arbitrary deviations from equilibrium, as well as to test the validity of the fluctuation theorem for this model. 

## 2. Ehrenfest–Klein Model

The essence of the model is as follows [18]. Let a system of *N* balls consist of two subsystems (boxes) A and B containing *n* and *N*–*n* balls, respectively. A ball is randomly transferred either from A to B with a probability *p* or from B to A with a probability *q*. Obviously, there is a nonzero probability that any transfer does not occur at any time (at each discrete step) (with the probabilities 1–*p* and 1–*q* for the subsystems A and B, respectively).

The total number of balls in both subsystems A and B is fixed; therefore, it is sufficient to follow the number of balls in one of two subsystems, e.g., the subsystem A. The transition from *n*_0_ to *n_τ_* balls in the subsystem A in τ discrete steps is denoted as (*n*_0_→*n*_τ_; τ). The transition can be implemented in various ways; thus, the total probability is the sum of the probabilities of individual trajectories. Under the assumption that the subsystem A is “prepared” in advance with the number of balls *n*_0_, this relation can be written as

(1) $$P\left(n_{0}\to n_{\tau };\tau \right)=\sum_{j}\omega_{j}(n_{1}^{j}|n_{0})\cdot \omega_{j}(n_{2}^{j}|n_{1}^{j})\cdot \ldots \cdot \omega_{j}(n_{\tau }|n_{m-1}^{j}),$$

where *j* is the index of a particular trajectory and *ω* are the transition probabilities:

(2) $$\omega_{j}\left(n_{\tau +1}^{j}|n_{\tau }^{j}\right)=\{\begin{array}{c} q\frac{N-n_{\tau }^{j}}{N},\;n_{\tau +1}^{j}=n_{\tau }^{j}+1,\\ p\frac{n_{\tau }^{j}}{N},\;n_{\tau +1}^{j}=n_{\tau }^{j}-1,\\ 1-q\frac{N-n_{\tau }^{j}}{N}-p\frac{n_{\tau }^{j}}{N},\;n_{\tau +1}^{j}=n_{\tau }^{j}. \end{array}$$

An exact solution of this model is known [18]. This solution leads in particular to the following average number of balls in the subsystem А in τ steps:

(3) $$\langle n\left(\tau \right)\rangle =n_{eq}+\left(n_{0}-n_{eq}\right){\left(1-\frac{p+q}{N}\right)}^{\tau },$$

where

(4) $$n_{eq}=\frac{q}{p+q}N\;.$$

If the number of balls in the system is large (*N* >>1), using the known relation $\underset{x\to 0}{\lim}{\left(1+x\right)}^{1/x}=e\;$, we obtain

$${\left(1-\frac{p+q}{N}\right)}^{\tau }={\left(1-\frac{p+q}{N}\right)}^{\left(-\frac{N}{p+q}\right)\cdot \left(-\frac{p+q}{N}\right)\tau }\approx e^{-\frac{p+q}{N}\tau }$$

and Equation (3) is represented in the form

(5) $$\langle n\left(\tau \right)\rangle =n_{eq}+\left(n_{0}-n_{eq}\right)e^{-\frac{p+q}{N}\tau }.$$

Below, we omit angle brackets but remember that we deal with average values.

## 3. Physicochemical System Consistent with the Ehrenfest–Klein Model: Thermodynamic Consideration

We consider a physicochemical system mathematically based on the game model described above. Let *N* particles be distributed between subsystems A (with *n* particles) and B (with *N*–*n* particles). The subsystems are at a constant temperature *T* and the total number of particles *N* is constant. Let the particles in each of the subsystems form an ideal gas and the number of particles in the subsystems at any time is much larger than unity. Inside any subsystem, the ideal gas is in thermal equilibrium, whereas the subsystems themselves are not in equilibrium with each other and have different energies *ε*_A_ and *ε*_B_.

We assume that a certain analog of a chemical reaction (or phase transformation) occurs between the subsystems, resulting in the transfer of particles from the subsystem A to the subsystem B. For a given arbitrary initial distribution of particles, they will be redistributed between the subsystems A and B until equilibrium values are reached. This flux of particles generates an energy flux between the system and thermostat because the energy Δε should be obtained from the thermostat for the transition of an individual particle to a higher energy state (A←B). On the contrary, the transition to a lower energy state (A→B) is accompanied by the transfer of the energy Δ*ε* from the system to the thermostat. The transfer of *dn* particles of the system from one subsystem to the other results in a change in the entropy *dS* [10]:

(6) $$TdS=\left(\varepsilon_{A}-\varepsilon_{B}\right)dn-\left(\mu_{A}-\mu_{B}\right)dn,$$

where *μ_i_* is the chemical potential. 

The transition probabilities *p* and *q* are introduced as

(7) $$p=qe^{\frac{\varepsilon_{A}-\varepsilon_{B}}{T}},$$

where it is assumed that *ε*_A_ > *ε*_B_ and the temperature is measured in energy units. 

Relation (7) expresses the fact that to pass from the subsystem B to the subsystem A (unlike the reverse transfer A→B), a particle should overcome an additional energy barrier Δ*ε* = *ε*_A_ – *ε_B_*. The probabilities *p* and *q* have the meaning of the rate constants of the direct A→B and reverse A←B reactions, respectively. The chemical potentials for the ideal subsystems under consideration are determined in terms of the partial pressures of the components and, consequently, have the form [10]:

(8) $$\begin{array}{l} \mu_{A}=\mu_{0A}\left(T,\varepsilon_{A}\right)+T\ln n,\\ \mu_{B}=\mu_{0B}\left(T,\varepsilon_{B}\right)+T\ln\left(N-n\right), \end{array}$$

where *μ_0_*_A_(*T*,*ε*_A_) and *μ_0_*_B_(*T*,*ε*_B_) are the chemical potentials in the standard state, which satisfy the relation [10].

(9) $$\mu_{0A}\left(T,\varepsilon_{A}\right)-\mu_{0B}\left(T,\varepsilon_{B}\right)=T\ln\frac{p}{q}.$$

Using Equations (8) and (9), we represent the difference between the chemical potentials of the A⇄B processes in the form:

(10) $$\Delta \mu =\mu_{A}-\mu_{B}=T\ln\frac{pn}{q\left(N-n\right)}.$$

At equilibrium in the system, Δ*μ* = 0 and, according to Equation (10), *pn_eq_* = *q*(*N* – *n_eq_*). Consequently, the equilibrium concentration *n_eq_* satisfies the relation (4). Using Equation (10), we rewrite (6) in the form

(11) $$\frac{dS}{d\tau }=-\ln\frac{pn}{q\left(N-n\right)}\frac{dn}{d\tau }+\ln\frac{p}{q}\frac{dn}{d\tau }.$$

It is well known that the entropy change rate in the system *dS/d*τ can be represented in the form [6,10]:

(12) $$\frac{dS}{d\tau }=\Sigma +J_{s},$$

where *J_s_* is the reversible part of the entropy increment caused by the energy flux through the boundaries of the system and Σ is the part of the increment caused by the irreversible processes inside the system, which is usually referred to as the entropy production.

Since change in the number of particles in the subsystem is accompanied by the energy exchange *T*ln(*p*/*q*) per particle with the environment (see Equation (7)), the second term in Equation (11) characterizes the entropy flux exchange between the system and environment when the number of particles in the subsystem А is changed by *dn*. This term can be both positive and negative. The first term in Equation (11) is the product of the difference between the chemical potentials of the subsystems and change in the number of particles. It is easily seen that this term is always nonnegative. Indeed, if the transition rate from the subsystem A to the subsystem В is higher than the reverse rate, the logarithm appears to be positive and the number of particles in the subsystem A decreases (*dn/d*τ < 0); on the contrary, if the transition rate from the subsystem A is lower than the reverse transition rate, the logarithm is negative, but *dn/d*τ > 0. For the mentioned reasons, the first term in Equation (11) can be called the entropy production in the system [10]. Thus,

(13) $$J_{s}=\ln\frac{p}{q}\frac{dn}{d\tau },$$

(14) $$\Sigma =-\ln\frac{pn}{q\left(N-n\right)}\frac{dn}{d\tau }.$$

## 4. Time Dependence of the Entropy of the System at the Evolution to the Equilibrium State

Using Equations (4), (5), (13) and (14), we write the entropy flux in the form

(15) $$J_{S}\left(\tau \right)=-\frac{\left(p+q\right)}{N}\left(n_{0}-n_{eq}\right)e^{-\frac{p+q}{N}\tau }\ln\left(\frac{N-n_{eq}}{n_{eq}}\right)$$

and the entropy production in the system under consideration as

(16) $$\Sigma \left(\tau \right)=\frac{\left(p+q\right)}{N}\left(n_{0}-n_{eq}\right)e^{-\frac{p+q}{N}\tau }\left[\ln\left(\frac{1+\frac{\left(n_{0}-n_{eq}\right)}{n_{eq}}e^{-\frac{p+q}{N}\tau }}{1+\frac{\left(n_{eq}-n_{0}\right)}{N-n_{eq}}e^{-\frac{p+q}{N}\tau }}\right)\right].$$

Relations (15) and (16) were derived from the average numbers of particles in the subsystems at each time (5). An expression similar to Equation (16) can be obtained by considering a chemical reaction whose rate is proportional to the concentration of a reagent [10]. In this work, the derivation is based on a particular statistical model rather than on a phenomenological approach [10]. Another statistical approach resulting in Equation (16) can be found in [19], but presentation in that work was not appropriate and did not contain any proof.

**Figure 1:**
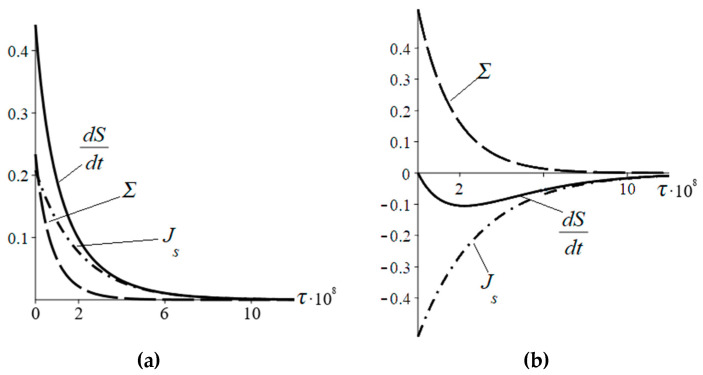
(Solid line) Total entropy change and its components: (dash-dotted line) entropy flux *J_s_*(*τ*) and (dashed line) entropy production Σ(*τ*). (**a**) *n*_0_ = 0.1 × 10^8^, (**b**) *n*_0_ = 1 × 10^8^. *p* = 0.8, *q* = 0.2, *n_eq_* = 0.4 × 10^8^ (see Equation (4)), *N* = 2 × 10^8^.

Figure 1 shows the time dependences of the flux (15) and entropy production (16), as well as their sum, i.e., the total entropy change. As seen, when *ε*_A_ > *ε*_B_, an increase in the number of particles in the subsystem A in time results in the inflow of the entropy into the system *J_s_* > 0 at *n*_0_ < *n_eq_*, whereas a decrease in the number of particles in the subsystem A in time results in the outflow of the entropy from the system *J_s_* < 0 at *n*_0_ > *n_Equation_*. According to Figure 1, the entropy production is always positive and tends to zero near equilibrium (*t* → ∞). 

The law of relaxation of the entropy production (16) is nonexponential. This expression contains an additional logarithmic factor. However, if the system is near equilibrium (either at the initial time, when *n*_0_ is near *n_eq_* or in the limit *t* → ∞), the argument of the logarithm is close to unity and the expansion of Equation (16) in a series gives

(17) $$\Sigma \left(\tau \right)=\frac{\left(p+q\right)}{N}{\left(n_{0}-n_{eq}\right)}^{2}\left(\frac{N}{n_{eq}\left(N-n_{eq}\right)}\right)e^{-\frac{2\left(p+q\right)}{N}\tau }.$$

**Figure 2:**
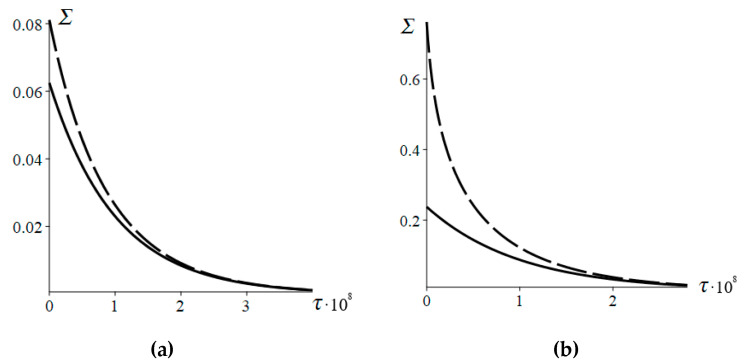
Entropy production according to Equation (16) (solid line) and Equation (17) (dashed line) versus the time *τ*. (**a**) *n*_0_ = 0.2 × 10^8^, (**b**) *n*_0_ = 0.01 × 10^8^. *N* = 2 × 10^8^, *p* = 0.8, *q* = 0.2, *n_eq_* = 0.4 × 10^8^.

Figure 2 shows the time dependence of the entropy production (16) and its approximate expression (17). It is seen that the approximate expression (17) well describes the entropy production (16) if the initial deviation from equilibrium is small. On the contrary, if the initial deviation from equilibrium is large, the approximate expression (17) is inconsistent with (16) at small times.

The entropy production is usually represented as the product of the thermodynamic forces *X* and fluxes *J* [6]. According to Equations (14) and (16) we have

(18) $$J\left(\tau \right)=\frac{dn}{d\tau }=-\frac{\left(p+q\right)}{N}\left(n_{0}-n_{eq}\right)e^{-\frac{p+q}{N}\tau },$$

(19) $$X=-\ln\frac{pn}{q\left(N-n\right)}=-\ln\left(\frac{1+\frac{\left(n_{0}-n_{eq}\right)}{n_{eq}}e^{-\frac{p+q}{N}\tau }}{1+\frac{\left(n_{eq}-n_{0}\right)}{N-n_{eq}}e^{-\frac{p+q}{N}\tau }}\right).$$

It follows from Equations (18) and (19), that the relation between the force and flux *X*(*J*) is strongly nonlinear:

(20) $$X\left(J\right)=\ln\frac{1+\frac{N}{\left(N-n_{eq}\right)\left(p+q\right)}J}{1-\frac{N}{n_{eq}\left(p+q\right)}J}.$$

The relation becomes linear near equilibrium (*t*→∞; *J*→0). Indeed, the expansion of the logarithm in Equation (20) into a Taylor series in a small flux yields

(21) $$\begin{array}{c} X\left(J\right)=\ln\left(1+\frac{N}{\left(N-n_{eq}\right)\left(p+q\right)}J\right)-\ln\left(1-\frac{N}{n_{eq}\left(p+q\right)}J\right)\approx \frac{N}{\left(N-n_{eq}\right)\left(p+q\right)}J+\frac{N}{n_{eq}\left(p+q\right)}J=\\ =\frac{N^{2}}{\left(p+q\right)\left(N-n_{eq}\right)n_{eq}}J=\frac{p+q}{pq}J. \end{array}$$

As seen from the linearized relation, the parameter (*p* + *q*)/(*pq*) is an analog of the kinetic coefficient in the model under consideration.

**Figure 3:**
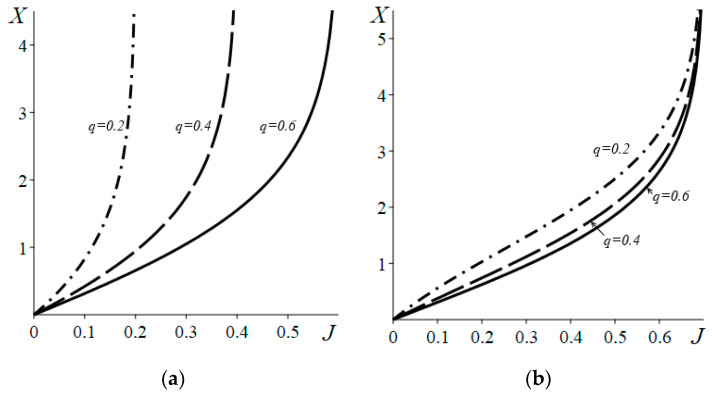
Relation between the thermodynamic force and flux *X*(*J*) for the system with *N* =2 × 10^8^ and *p* = 0.7. (**a**) Initial number of particles in the subsystem B is larger than that in the subsystem A (*X* > 0, *J* > 0); (**b**) the initial number of particles in the subsystem A is larger than that in the subsystem B (*X* < 0, *J* < 0, the axes present absolute values).

Examples of *X*(*J*) dependences are shown in Figure 3. As seen, the dependences near equilibrium (*J→*0) are well described by a linear law, but the dependence far from equilibrium becomes nonlinear. The figure shows that the thermodynamic force tends to infinity when the flux approaches a certain *J*_max_ value. The *J*_max_ values can be found from Equations (4) and (20), by equating the argument of the logarithm to zero. As a result, for the case Figure 3a, we obtain

(22) $$J_{\max}=\frac{n_{eq}\left(p+q\right)}{N}=q,$$

whereas for the case of Figure 3b, we have

(23) $$\left|J_{\max}\right|=\frac{\left(N-n_{eq}\right)\left(p+q\right)}{N}=p.$$

Thus, the Ehrenfest–Klein system implies a fundamental restriction on the maximum possible flux *J*_max_, which is due to the finiteness and constancy of the total number of particles *N* in the system. A picture similar to Figure 3 is also observed in the case of *p* < *q*.

## 5. Test of the Validity of the Fluctuation Theorem

Fluctuation theorem connects measure of dissipation (dissipation functional) of direct and reverse process. There are several formulations of the fluctuation theorem, which are different in form but common in idea. Originally, fluctuation relation is obtained for a thermostatted shear-driven fluid using numerical simulations by Evans et al. [1,3]. Fluctuation theorem was first proven for a large class of systems using concepts from chaotic dynamics by Gallavotti and Cohen. Later the fluctuation theorem was extended for systems with diffusive and Langevin dynamics, Markovian stochastic systems, etc. [2,4]. One of the widely known formulations is the transient fluctuation theorem (TFT) which was found by Evans and Searles [1]. TFT applies to systems evolving over finite time *τ* between two arbitrary states that are not necessarily required to be in equilibrium. According to the transient fluctuation theorem in simplest form applicable for further consideration [1,3],

(24) $$\frac{P\left(\Omega \right)}{P\left(-\Omega \right)}=e^{\Omega \tau },$$

where *P*(± Ω) is the probability of observing a trajectory with a dissipation functional equal to ± Ω. A positive value characterizes the direction of the process to equilibrium, whereas a negative value indicates the development of the system to a larger deviation from equilibrium. The transient fluctuation theorem is often considered as a generalization of the second law of thermodynamics because it is applicable to the systems evolving in time at any deviation from equilibrium.

A specific form of the dissipation functional is determined by the properties of the system under study. Under certain assumptions (such as a large number of particles, the presence of local equilibrium, small nonequilibrium, etc.), it is supposed that the dissipation functional is reduced to the thermodynamic entropy production [3]. However, this is not necessarily true. In particular, it was demonstrated in [20] that the identification of the dissipation functional with the thermodynamic entropy production is incorrect in the case of the Schlӧgl model under the conditions of local equilibrium and a large number of particles. For a number of systems described by the nonlinear Langevin equation, the authors of [21] also showed that the identification of the dissipation functional with the entropy production in the transient fluctuation theorem leads to invalid results. In this work, we analytically test the possibility of identifying the dissipation functional Ω with the thermodynamic entropy production in the transient fluctuation theorem in application to the Ehrenfest–Klein model. 

We consider the variation of the number of particles in the subsystem А from *n*_1_ to *n*_2_ in a time interval *τ*, where *n*_1_ and *n*_2_ correspond to two locally equilibrium states and satisfy Equation (5). Let *n*_1_ < *n*_2_ < *n*_eq_ for definiteness; i.e., as the number of particles in the subsystem А increases, the system approaches equilibrium. For this model, Equation (24) can be represented in the form

(25) $$\ln\frac{P\left(n_{1}\to n_{2};\tau \right)}{P\left(n_{2}\to n_{1};\tau \right)}=\int_{0}^{\tau }\Sigma \left(t\right)dt,$$

where the numerator and denominator of the left-hand side include the probabilities of the process approaching the system to equilibrium and deviating it from equilibrium, respectively.

We transform the right-hand side of Equation (25). According to Equation (14),

(26) $$\int_{0}^{\tau }\Sigma \left(t\right)dt=-\int_{n_{1}}^{n_{2}}\ln\frac{pn}{q\left(N-n\right)}dn=N\ln\frac{N-n_{1}}{N-n_{2}}+n_{2}\ln\frac{n_{eq}\left(N-n_{2}\right)}{\left(N-n_{eq}\right)n_{2}}+n_{1}\ln\frac{\left(N-n_{eq}\right)n_{1}}{n_{eq}\left(N-n_{1}\right)}.$$

We now calculate the left-hand side of Equation (25). In one step, only one elementary event occurs; therefore, Equation (1) always contains exactly τ factors that are the probabilities (rates) of elementary transitions. The transition process is a chain of locally equilibrium states. According to Equation (1), only some possible trajectories at a given τ value can ensure the necessary transition Δ*n* = *n*_2_ – *n*_1_. Moreover, for the transition probabilities in τ steps to be nonzero, the inequality *τ* ≥ Δ*n* should be satisfied. Let τ = Δ*n* + ξ, where ξ is some non-negative integer. The total number of steps τ can be represented as the sum of Δ*n* + *h* steps in which the transitions from B to A occur, *h* steps in which the transitions from A to B occur, and ξ – 2*h* steps in which the numbers of particles in the subsystems do not change. Since ξ – 2*h* ≥ 0, *h* can be from zero to *h*_max_ = int(ξ/2) (where int(*x*) means the integer part of the number *x*).

We determine the possible number of trajectories corresponding to the (*n*_1_→*n*_1_ + Δ*n*; τ) transitions. The entire set of trajectories can be divided into *h*_max_ + 1 groups specified by the *h* values. Within one group, the trajectories differ only in the sequence of elementary events from Equation (1). Thus, the number of trajectories in a group with a certain *h* value will be determined by the number of all possible ways to combine all the elementary transitions (ignoring the steps at which the number of particles does not change), i.e., Δ*n* + *h* steps with transitions from B to A and *h* steps with transitions from A to B:

(27) $$\left(\begin{array}{c} \Delta n+\xi \\ \Delta n+2h \end{array}\right)=\frac{\left(\Delta n+\xi \right)!}{\left(\Delta n+2h\right)!\left(\xi -2h\right)!}.$$

The total number of trajectories is given by the sum

(28) $$M=\sum_{h=0}^{h_{\max}}\frac{\left(\Delta n+\xi \right)!}{\left(\Delta n+2h\right)!\left(\xi -2h\right)!}.$$

In the Ehrenfest–Klein model, a transition from any state with a certain number of particles to any other one is possible: “forbidden” trajectories are absent, and each trajectory corresponds to the conjugate one, in which all the same states are passed in τ steps, but in the reverse order. Consequently, the total numbers of trajectories corresponding to the (*n*_1_ → *n*_1_ + Δ*n*; τ) and (*n*_1_ + Δ*n* → *n*_1_; τ) transitions are equal to each other.

**Figure 4:**
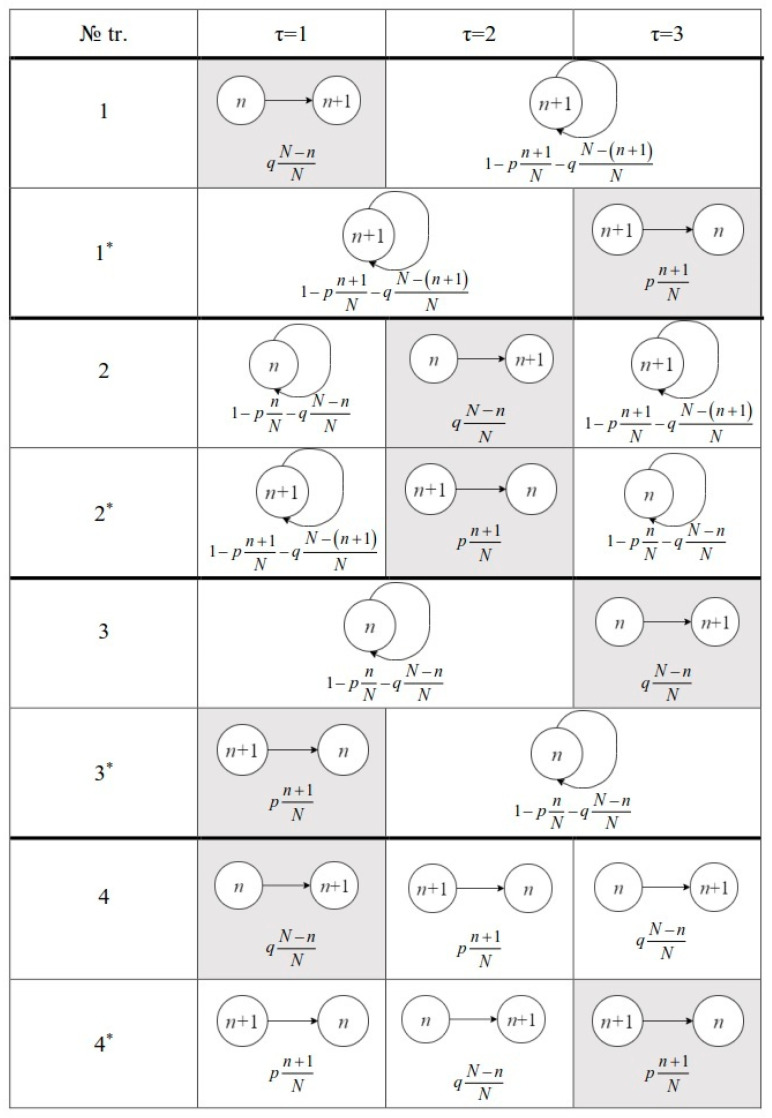
Example of the complete set of trajectories of the transition (*n*→*n* + 1; *τ* = 3) and the set of conjugate trajectories (marked by *) for the reverse transition (*n* + 1→*n*; *τ* = 3). The schemes of elementary transitions and their probabilities are indicated for each trajectory at every time instant. It is seen that each of the trajectories of a transition (direct or reverse) contains the identical part marked in white.

We now consider the structure of a single transition trajectory (*n*_1_→*n*_1_+Δ*n*; τ = Δ*n*+ξ). For clarity, we consider the example in Figure 4. In Equation (1), individual factors can be combined into two groups according to their value. The first group consists of Δ*n* factors that correspond to a sequential transition through the states *n*_1_, *n*_1_+1, *n*_1_+2, …, *n*_1_+Δ*n* in Δ*n* steps, i.e., (*n*_1_→*n*_1_+Δ*n*; Δ*n*). This group of factors is the same for any trajectory (*n*_1_→*n*_1_+Δ*n*; *τ* = Δ*n*+ξ). In Figure 4, such factors are shown in gray. The second group includes the remaining elementary transitions, each of which is "compensated" by the reverse transition; as a result, they return the system to its initial state rather than transfer it to new states. In Figure 4, such factors are indicated in white. A similar consideration is applicable to the reverse transition (*n*_1_+Δ*n*→*n*_1_; τ = Δ*n*+ξ). Since each elementary transition from the second group of states is necessarily accompanied by a reverse one, the entire set of such transitions is not sensitive to the direction of the global transition (in the forward or reverse direction). They are also fully compensated, and, in fact, return the system to its initial state. Therefore, the factors from the second groups for the direct *j* and reverse j^*^ trajectories completely coincide with each other (this is clearly seen in Figure 4) and they can be taken out of the summation sign and canceled when writing the left-hand side of the fluctuation theorem.

As a result, using Equation (1), the left-hand side of Equation (25) can be written in the form

(29) $$\ln\frac{P\left(n_{1}\to n_{1}+\Delta n;\tau =\Delta n+\xi \right)}{P\left(n_{1}+\Delta n\to n_{1};\tau =\Delta n+\xi \right)}=\ln\frac{\sum_{j=1}^{M}\overset{\Delta n\;\mathrm{factors}}{\overbrace{\left\{\mathrm{group}\;1\right\}}}\times \overset{\xi (j)\;\mathrm{factors}}{\overbrace{{\left\{\mathrm{group}\;2\right\}}_{j}}}}{\sum_{j^{*}=1}^{M}\underset{\Delta n\;\mathrm{factors}}{\underbrace{{\left\{\mathrm{group}\;1\right\}}^{*}}}\times \underset{\xi (j^{*})\;\mathrm{factors}}{\underbrace{{\left\{\mathrm{group}\;2\right\}}_{j^{*}}^{*}}}},$$

where:

(30) $$\begin{array}{l} \left\{\mathrm{group}\;1\right\}={\left(\frac{q}{N}\right)}^{\Delta n}\prod_{k=n_{1}}^{n_{1}+\Delta n-1}\left(N-k\right),\\ {\left\{\mathrm{group}\;1\right\}}^{*}={\left(\frac{p}{N}\right)}^{\Delta n}\prod_{k^{*}=n_{1}+1}^{n_{1}+\Delta n}k^{*},\\ {\left\{\mathrm{group}\;2\right\}}_{j}\equiv {\left\{\mathrm{group}\;2\right\}}_{j^{*}}^{*}. \end{array}$$

After simple transformations of Equations (29) and (30), we have

(31) $$\ln\frac{P\left(n_{1}\to n_{1}+\Delta n;\tau =\Delta n+\xi \right)}{P\left(n_{1}+\Delta n\to n_{1};\tau =\Delta n+\xi \right)}=\ln\left(\frac{q\left(N-n_{1}\right)\times q\left(N-\left(n_{1}+1\right)\right)\times \ldots \times q\left(N-\left(n_{1}+\Delta n\right)\right)}{p\left(n_{1}+\Delta n\right)\times p\left(n_{1}+\left(\Delta n-1\right)\right)\times \ldots \times p\left(n_{1}+1\right)}\right).$$

The right-hand-hand side of Equation (31) can be represented in the shorter form

(32) $$\ln\left({\left(\frac{q}{p}\right)}^{\Delta n}\cdot \frac{\left(N-n_{1}\right)!}{\left(N-\left(n_{1}+\Delta n\right)\right)!}\cdot \frac{n_{1}!}{\left(n_{1}+\Delta n\right)!}\right).$$

Assuming that the number of particles in all considered stages of evolution is much larger than unity, we transform Equation (32) using the Stirling formula and Equation (4):

(33) $$\Delta n\ln\frac{q}{p}+\left(N-n_{1}\right)\ln\left(N-n_{1}\right)-\left(N-\left(n_{1}+\Delta n\right)\right)\ln\left(N-\left(n_{1}+\Delta n\right)\right)+n_{1}\ln n_{1}-\left(n_{1}+\Delta n\right)\ln\left(n_{1}+\Delta n\right).$$

After a series of simple transformations, Equation (33) exactly coincides with Equation (26).

Thus, at the introduced restrictions (a large number of particles in the subsystems and the hypothesis of local equilibrium at each step), the transient fluctuation theorem turns out to be valid for the Ehrenfest–Klein model if the thermodynamic entropy production is used as the dissipation functional.

## 6. Conclusions

Nonequilibrium thermodynamic relations have been derived for the simplest physicochemical system considered within the classical Ehrenfest–Klein model. A nonlinear relation between the thermodynamic flux (reaction rate) and force (affinity) valid for an arbitrary deviation from equilibrium has been obtained. A nonexponential time dependence of the entropy production when the system approaches equilibrium is an interesting result of this work. The validity of the transient fluctuation theorem, where the thermodynamic entropy production is used as a dissipation functional, has been tested. The results obtained will be useful for the development of nonlinear nonequilibrium thermodynamics, which is still incomplete, unlike linear thermodynamics. The derived relations could be applied to different systems with two energy levels (two-level systems). For example, consideration of the interaction of radiation with matter and membrane transport processes (in particular, biological ones) is very promising.
